# Fermented Oyster Extract Prevents Ovariectomy-Induced Bone Loss and Suppresses Osteoclastogenesis

**DOI:** 10.3390/nu11061392

**Published:** 2019-06-21

**Authors:** Hye Jung Ihn, Ju Ang Kim, Soomin Lim, Sang-Hyeon Nam, So Hyeon Hwang, Jiwon Lim, Gi-Young Kim, Yung Hyun Choi, You-Jin Jeon, Bae-Jin Lee, Jong-Sup Bae, Yeo Hyang Kim, Eui Kyun Park

**Affiliations:** 1 Institute for Hard Tissue and Biotooth Regeneration (IHBR), Kyungpook National University, Daegu 41940, Korea; hjpihn@hanmail.net; 2 Department of Oral Pathology and Regenerative Medicine, School of Dentistry, IHBR, Kyungpook National University, Daegu 41940, Korea; kkangjin5@hanmail.net (J.A.K.); friendship1240@hanmail.net (S.L.); aay0805@naver.com (S.-H.N.); shhwang1010@naver.com (S.H.H.); jiwonparadise@hanmail.net (J.L.); 3 Department of Marine Life Sciences, Jeju National University, Jeju 63243, Korea; immunkim@jejunu.ac.kr (G.-Y.K.); youjinj@jejunu.ac.kr (Y.-J.J.); 4 Department of Biochemistry, College of Oriental Medicine, Dong-Eui University, Busan 47227, Korea; choiyh@deu.ac.kr; 5 Marine Bioprocess Co., Ltd., Busan 46048, Korea; hansola82@hanmail.net; 6 College of Pharmacy, Research Institute of Pharmaceutical Sciences, Kyungpook National University, Daegu 41566, Korea; baejs@knu.ac.kr; 7 Department of Pediatrics, School of Medicine, Kyungpook National University, Daegu 41944, Korea; kimyhmd@knu.ac.kr

**Keywords:** osteoclast, ovariectomy, fermented oyster extract, bone loss

## Abstract

There is growing interest in bioactive substances from marine organisms for their potential use against diverse human diseases. Osteoporosis is a skeletal disorder associated with bone loss primarily occurring through enhanced osteoclast differentiation and resorption. Recently, we reported the anti-osteoclastogenic activity of fermented Pacific oyster (*Crassostrea gigas*) extract (FO) in vitro. The present study focused on investigating the anti-osteoporotic efficacy of FO in bone loss prevention in an experimental animal model of osteoporosis and elucidating the mechanism underlying its effects. Oral administration of FO significantly decreased ovariectomy-induced osteoclast formation and prevented bone loss, with reduced serum levels of bone turnover biomarkers including osteocalcin and C-terminal telopeptide fragment of type I collagen C-terminus (CTX). FO significantly suppressed receptor activator of nuclear factor-κB ligand (RANKL)-induced differentiation of bone marrow-derived macrophages (BMMs) into osteoclasts and attenuated the induction of osteoclast-specific genes required for osteoclastogenesis and bone resorption. Furthermore, FO inhibited RANKL-mediated IκBα and p65 phosphorylation in BMMs. Taken together, these results demonstrate that FO effectively suppresses osteoclastogenesis in vivo and in vitro, and that FO can be considered as a potential therapeutic option for the treatment of osteoporosis and osteoclast-mediated skeletal diseases.

## 1. Introduction

Osteoporosis is a metabolic skeletal disorder with significant public health ramifications given its increasing prevalence, especially in elderly people [1]. Aging, smoking, nutrition, and menopause are potential causative factors for developing osteoporosis [2]. With osteoporosis, diminished bone density and reduced strength contribute to increased fracture risk. The loss of bone mass and strength is closely associated with dysregulated bone remodeling; in healthy individuals, this process comprises the removal of old bone by osteoclasts and replacement with new bone at the same site by osteoblasts and plays a central role in maintaining bone integrity and mineral homeostasis [3,4]. As bone removal surpasses deposition, bone loss and architectural deterioration of skeletal tissue occur, which can lead not only to osteoporosis but also several other bone diseases such as periodontitis and rheumatoid arthritis [5,6,7]. Various antiresorptive drugs including bisphosphonates, denosumab, and calcitonin are available for the treatment of osteoporosis via their ability to protect against osteoclastic resorption and preserve bone mass [8]. Although these pharmacological treatments have positive effects on skeletal health, concerns regarding side effects such as osteonecrosis of the jaw in bisphosphonate and denosumab and an increased risk of liver cancer in calcitonin have emerged, which have led to increased interest in the discovery of alternative agents capable of suppressing osteoclastogenesis [9,10,11].

Previous studies have shown that natural products including diverse extracts and compounds have regulatory activities and potential therapeutic utility through their antioxidant, anticancer, antimicrobial, and anti-inflammatory properties [12]. In particular, marine products have attracted attention as a new source for drug discovery, as marine organisms produce and store a variety of natural bioactive substances for protection and adaptation to harsh environmental conditions [13]. In fact, it has been demonstrated that extracts or bioactive compounds from marine organisms can exert profound effects on bone metabolism by enhancing osteoblastogenesis and suppressing osteoclast function, which are considered to be especially promising for the treatment of bone diseases such as osteoporosis [14]. Such osteoprotective effects of marine natural products are considered to be partially associated with their antioxidant or anti-inflammatory activities. Uchiyama and Yamaguchi reported that the water-solubilized *Sargassum horneri* extract stimulates osteoblastic bone formation and suppresses osteoclastic bone resorption, exhibiting both osteogenic and anti-osteoclastogenic properties [15]. In addition, the extract of *Padina Pavonica* was shown to improve calcium uptake and is currently marketed as a dietary supplement to prevent osteoporosis [16]. 

Among marine organisms, oysters contain diverse bioactive compounds with antioxidant activity [17]. A recent study on bone growth revealed that both oyster extract and taurine, a main component of oysters, can improve various bone parameters including bone volume/tissue volume, trabecular thickness, and trabecular number and can increase growth plate thickness in an in vivo mouse model [17]. N16 found in the nacreous layer of *Pinctada fucata*, a pearl oyster, has been shown to impair receptor activator of nuclear factor-κB ligand (RANKL)-induced osteoclatogenesis and osteoclastic bone resorption [18]. More recently, we evaluated the effect of fermented oyster extract (FO) on osteoclast differentiation using RAW 264.7 cells, a monocytic cell line, and found that FO can effectively suppress osteoclast formation via reactive oxygen species (ROS) scavenging [19]. 

The aim of the present study was to clarify the in vivo effect of FO on bone loss in ovariectomized (OVX) mice as a mouse model of osteoporosis and elucidate the mechanisms underlying the inhibition of RANKL-mediated osteoclast differentiation using primary murine macrophages.

## 2. Results

### 2.1. Administration of FO Mitigates OVX-Induced Bone Loss in Vivo

**Figure 1:**
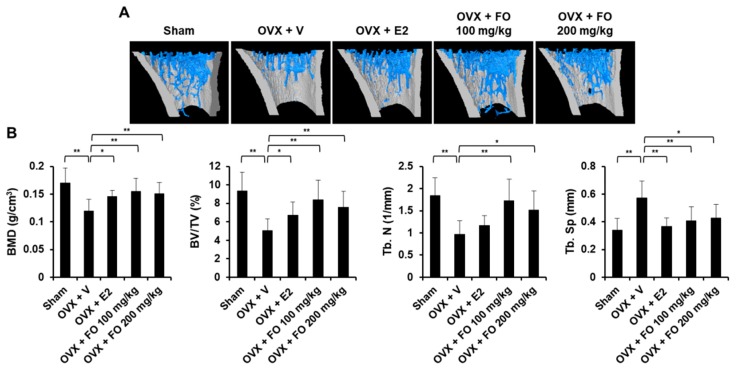
Fermented Pacific oyster (*Crassostrea gigas*) extract (FO) prevented ovariectomized (OVX)-induced bone loss in vivo. (**A**) 3D images of tibial trabecular bone in five groups: sham-operated + vehicle (Sham), OVX + vehicle (OVX + V), OVX + 17β-estradiol (E2, 10 μg/kg) (OVX + E2), OVX + low concentration of FO (OVX + FO 100 mg/kg), and OVX + high concentration of FO (OVX + FO 200 mg/kg). (**B**) Analysis of bone morphometric parameters including bone mineral density (BMD), bone volume per tissue volume (BV/TV), trabecular number (Tb. N), and trabecular separation (Tb. Sp) in each group. Values are the mean ± SD (*n* = 8). * *p* < 0.05, ** *p* < 0.01.

**Figure 2:**
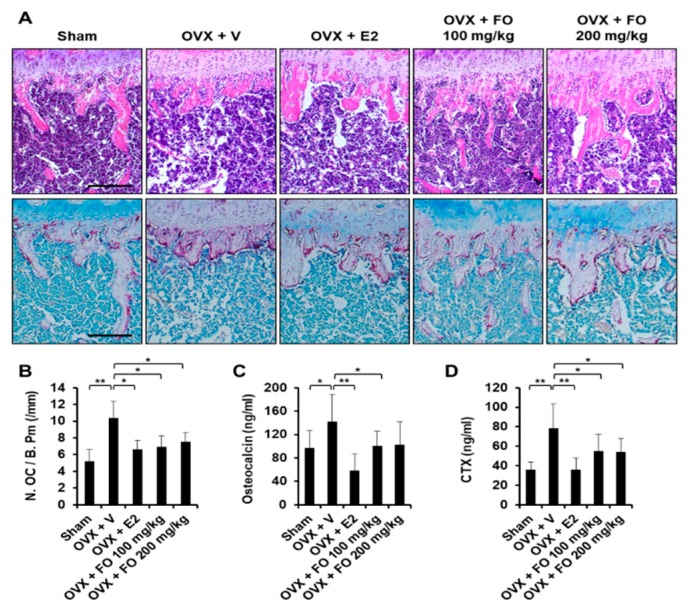
FO decreased OVX-induced osteoclast formation and reduced serum levels of osteocalcin and CTX. (**A**) Fixed tibias were decalcified, embedded in paraffin and sectioned. Tibial sections were stained with hematoxylin and eosin (H&E, upper panel) and tartrate-resistant acid phosphatase (TRAP, lower panel). (**B**) The number of osteoclasts per bone perimeter (N. OC/B. Pm) was quantified in the TRAP-stained section. Scale bar, 200 μm. (**C**) Quantification of serum osteocalcin concentrations (**D**) Quantification of CTX concentrations. Values are the mean ± SD (*n* = 8). * *p* < 0.05, ** *p* < 0.01.

To examine the in vivo effect of FO on bone loss, we applied FO in a mouse model of OVX-induced osteoporosis. The acclimatized mice were ether sham-operated or surgically ovariectomized. OVX mice were given vehicle, 17β-estradiol (E2, 10 μg/kg), or FO (100 or 200 mg/kg) once per day and sacrificed after 4 weeks of treatment. Mouse tibias were obtained, and bone morphometric parameters including bone volume per total volume (BV/TV), trabecular separation (Tb. Sp.), trabecular number (Tb. N.), and bone mineral density (BMD) in the proximal tibia were analyzed by μCT. As shown in μCT-scanned images, OVX mice exhibited a significant reduction in trabecular bone number and a lower uterus index (the ratio of uterine weight to body weight) compared with the sham-operated mice, indicating the success of ovariectomy (Figure 1A and Figure S1). BV/TV in the OVX group (5.04 ± 1.26%) was significantly decreased by 46% compared to the sham group (9.36 ± 2.01%), whereas administration of FO (100 mg/kg group: 8.38 ± 2.14%, and 200 mg/kg group: 7.59 ± 1.72%) attenuated trabecular bone loss (Figure 1B). In addition, trabecular separation in the low-dose FO group was significantly less while trabecular number was approximately 70% greater compared with the OVX group (Figure 1B). More significantly, FO-treated mice displayed increased BMD in tibias compared to OVX mice, indicating that FO was able to protect against bone loss (Figure 1B). E2, a positive control, significantly suppressed OVX-induced bone loss. To further confirm the in vivo effect of FO, we analyzed decalcified tibias by hematoxylin and eosin (H&E) and tartrate-resistant acid phosphatase (TRAP) staining. Consistent with the μCT analyses, FO treatment clearly increased trabecular density and decreased the number of TRAP-positive cells compared with the OVX group (Figure 2A,B). 

Serum concentrations of osteocalcin and CTX were significantly higher in ovariectomized mice compared with the sham group (Figure 2C,D), whereas OVX mice treated with 100 mg/kg FO showed approximately 30% lower osteocalcin and CTX levels compared with vehicle-treated OVX mice (Figure 2C,D).

### 2.2. FO Suppresses RANKL-Induced Osteoclast Formation in Vitro

**Figure 3:**
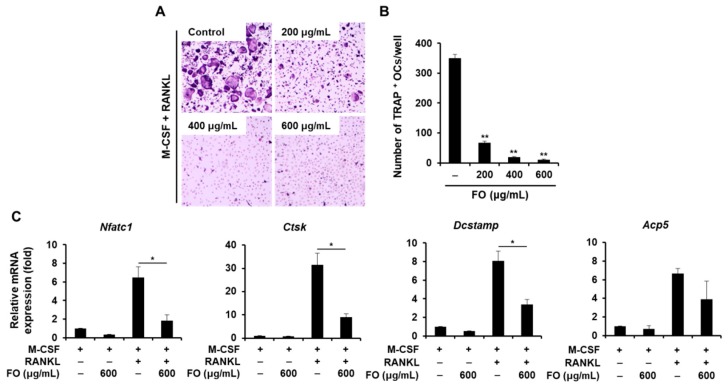
FO suppressed receptor activator of nuclear factor-κB ligand (RANKL)-mediated osteoclast formation. (**A**) Bone marrow-derived macrophages (BMMs) were cultured in the presence of M-CSF (10 ng/mL) and RANKL (20 ng/mL) with or without various concentrations of FO for 4 days. The cells were stained for TRAP expression. (**B**) The number of TRAP-positive multinuclear cells were quantified. (**C**) BMMs were cultured in the presence of M-CSF (10 ng/mL) or M-CSF (10 ng/mL) plus RANKL (20 ng/mL) with or without FO (600 μg/mL) for 4 days. Osteoclast-specific gene expression was evaluated by real-time PCR. +: treated, -: not treated. Values are the mean ± SD of three independent experiments. * *p* < 0.05, ** *p* < 0.01.

To investigate the effect of FO on RANKL-induced osteoclastogenesis in primary mouse osteoclast precursors, we treated BMMs with RANKL and M-CSF in the presence or absence of various concentrations of FO. As shown in Figure 3A, RANKL and M-CSF stimulated the differentiation of BMMs into TRAP-positive multinucleated osteoclasts in the vehicle-treated control, whereas the number of osteoclasts (TRAP-positive MNCs) was significantly decreased in the presence of FO, without any cytotoxicity (data not shown). In particular, FO at a concentration of 600 μg/mL led to strong inhibition (by approximately 93%) of osteoclast formation (Figure 3B). In contrast to its significant anti-osteoclastogenic effect, FO only weakly inhibited the bone-resorbing activity of mature osteoclasts (Figure S2), as the resorbed area in the presence of FO (600 μg/mL) was reduced by 41% compared to the vehicle-treated control.

### 2.3. FO Attenuates the Expression of Osteoclast-Specific Genes and the Formation of Actin Rings

**Figure 4:**
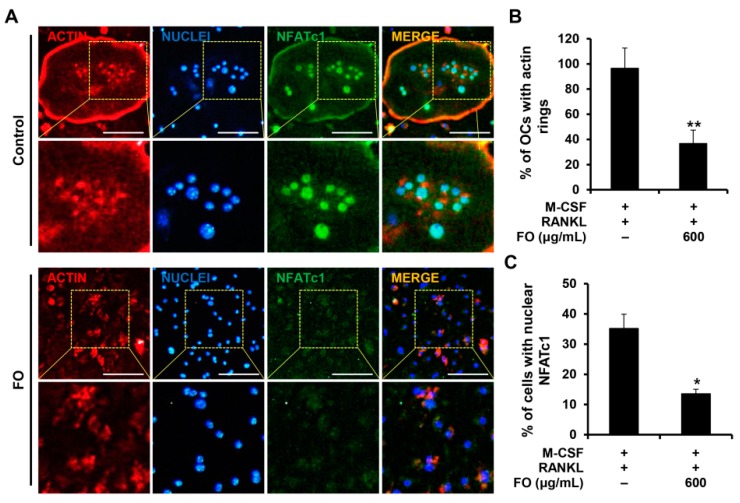
FO suppressed actin ring formation and nuclear localization of NFATc1. (**A**) BMMs seeded on glass coverslips were cultured in osteoclast-inducing medium with or without FO (600 μg/mL) for 4 days. Cells were fixed, and F-actin, nuclei, and NFATc1 were visualized by staining with rhodamine-conjugated phalloidin (red), DAPI (blue), and anti-NFATc1 antibody (green), respectively. Yellow dashed box: magnified region. Scale bar, 50 μm. The percentages of (**B**) osteoclasts forming actin rings and (**C**) cells with nuclear NFATc1 were quantified. +: treated, -: not treated. Values are the mean ± SD of three independent experiments. * *p* < 0.05, ** *p* < 0.01.

Next, we determined the effect of FO on osteoclast-related gene expression to further confirm its anti-osteoclastogenic property. NFATc1 is well known as the master regulator of osteoclastogenesis and bone resorbing function [20]. In response to M-CSF and RANKL, the mRNA levels of *Nfatc1* and its target genes including *Acp5*, *Ctsk*, and *Dcstamp* were all upregulated (Figure 3C). However, the expression of these osteoclast-specific genes and the degree of RANKL-induced nuclear localization of NFATc1 were clearly suppressed in the presence of FO (Figure 3C and Figure 4A,C). Consistent with the reduced *Dcstamp* expression, the number of cells with actin rings, a unique skeletal structure of osteoclasts, was also decreased with FO treatment (Figure 4A,B).

### 2.4. FO Suppresses the RANKL-Mediated NF-κB Signaling Pathway

**Figure 5:**
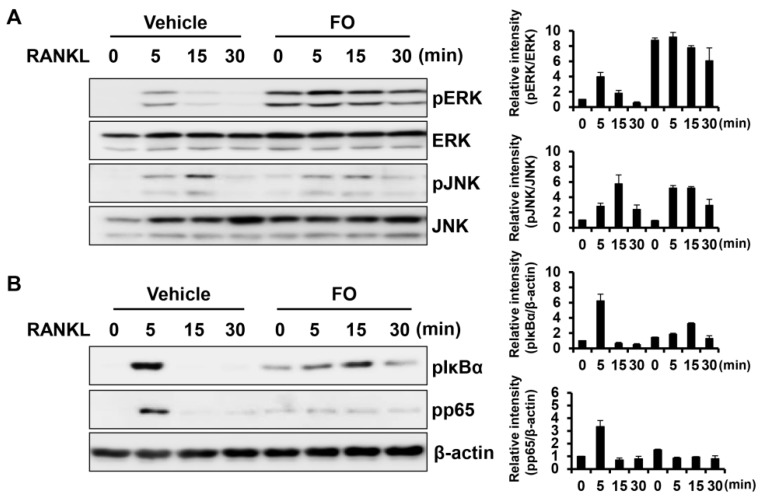
FO suppressed RANKL-mediated NF-κB pathway activation. (**A**,**B**) BMMs were pretreated with FO (600 μg/mL) or vehicle for 1 h and then stimulated with RANKL (50 ng/mL) for 0, 5, 15, and 30 min as indicated. Equal amounts of protein were subjected to western blot analysis with antibodies against the phosphorylated (p) forms of ERK, JNK, IκBα, and p65. β-actin served as the loading control. The images are representative of three independent experiments.

Activation of the MAPK and NF-κB signaling pathways mediated by RANKL plays an essential role in osteoclast differentiation and function [21,22]. To elucidate the molecular mechanism by which FO regulates osteoclastogenesis, these central signaling pathways responsible for osteoclast differentiation were examined. Serum-starved BMMs were pre-treated with FO or vehicle for 1 h followed by RANKL stimulation. As shown in Figure 5A, the phosphorylation levels of ERK was increased by pre-treatment with FO, but JNK was not affected by FO. In contrast, FO attenuated RANKL-induced phosphorylation of IκBα and p65 (Figure 5B).

## 3. Discussion

Vital components of marine shellfish have received interest for their various biological and pharmacological properties including antioxidant, anti-inflammatory, antimicrobial, and anticancer activities [23]. Among shellfish, oysters are one of the most prominent farm-raised marine shellfishes and contain appreciable amounts of proteins, minerals, and vitamins that are beneficial to human health. There is growing evidence describing the beneficial influence of oyster on bone strength. Oyster shell-derived calcium is available as a nutrition supplement to prevent osteoporosis and fracture [24]. However, these products have several issues associated with the large particle size and poor solubility of oyster shell powder that need improvement. We recently reported that fermented oyster extract (FO) exhibits anti-osteoclastogenic properties in RAW 264.7 cells, which led us to further investigate the in vivo effect of FO to evaluate its potential use as a therapeutic agent or a dietary supplement. In the current study, we found that FO significantly mitigated ovariectomy-induced bone loss and suppressed the differentiation of primary mouse BMMs into mature osteoclasts by attenuating the NF-κB signaling pathway, one of the major signaling cascades responsible for RANKL-induced osteoclastogenesis.

Estrogen deficiency contributes to the pathogenesis of osteoporosis, which is characterized by accelerating bone loss and increased fracture risk [25]. Ovariectomized animal models resembling the condition of postmenopausal osteoporosis are widely used in research seeking to develop alternative agents for the prevention and treatment of osteoporosis [26]. In the present study, tibial trabecular bone parameters including BMD and BV/TV were decreased by ovariectomy compared with the sham group, and the serum levels of bone formation (osteocalcin) and bone resorption (CTX) markers were higher in the OVX group, demonstrating increased bone turnover (Figure 1 and Figure 2). These results are in line with previous findings showing that a declining estrogen level accelerates bone remodeling rates, which eventually leads to bone loss [27]. However, administration of FO (100 or 200 mg/kg/day for 4 weeks) significantly ameliorated trabecular bone loss in ovariectomized mice by inhibiting osteoclast formation, and serum concentrations of OC and CTX were lower in the FO-treated groups than the OVX group, indicating the potential utility of FO as an anti-osteoporotic agent (Figure 1 and Figure 2). Although FO decreased the number of TRAP-positive osteoclasts and effectively preserved BMD and bone mass, its positive effect on osteoblast differentiation may not be excluded. A role of FO in osteoblast differentiation needs to be elucidated for a more complete understanding of its effects.

It is well established that RANKL acts as a key factor promoting osteoclast differentiation and that its binding to RANK triggers activation of the NF-κB pathway [28]. Upon RANKL stimulation, IκB is phosphorylated and subsequently degraded, which allows NF-κB to translocate to the nucleus to induce target gene transcription [29]. In vivo studies using genetically modified mutants support a critical role for NF-κB signaling in bone and osteoclast differentiation. NF-κB1/2 double knockout mice have severe osteopetrosis due to a defect in osteoclast formation [30]. p65^-/-^ chimeric mice have fewer TRAP-positive osteoclasts in response to RANKL injection [31]. In addition, osteoclast precursors from p65^-/-^ chimeric mice fail to differentiate into osteoclasts, as RelA/p65 is responsible for preventing cell death and facilitating progression to osteoclastgenesis, reflecting its antiapoptotic effect during osteoclast differentiation [31]. In this study, we observed that FO suppressed RANKL-mediated activation of the NF-κB pathway, as demonstrated by the attenuated phosphorylation of IκBα and p65 (Figure 5B). 

RANKL signaling eventually leads to the induction of NFATc1, a major transcription factor for osteoclastogenesis. NFATc1 controls the transcription of osteoclast-specific genes required for osteoclast differentiation and resorbing function including TRAP, cathepsin K, and DC-STAMP. Osteoclast-specific NFATc1 conditional knockout leads to an osteopetrotic phenotype resulting from defective osteoclast differentiation [32]. Lack of NFATc1 in embryonic stem cells results in the failure of osteoclast formation in response to RANKL, and ectopic expression of NFATc1 in these BMMs induces osteoclastogenesis, even in the absence of RANKL [20]. Consistent with previous studies, downregulation of *Nfatc1* by FO treatment reduced the induction of its target genes, ultimately preventing the differentiation of BMMs into osteoclasts (Figure 3). 

The major function of osteoclasts is to break down bone. The effect of FO on osteoclast function was investigated by resorption pit assay. We observed that FO weakly suppressed osteoclast bone resorbing function (Figure S2), indicating that FO may possess moderate antiresorptive activity.

The present study shows that oral administration of FO protects mice from OVX-induced osteoporosis by suppressing osteoclast formation. We also demonstrate that FO exhibits potent anti-osteoclastogenic and mild antiresorptive activities through its ability to impair the RANKL-induced NF-κB pathway. Thus, FO could be beneficial for preventing estrogen deficiency-induced bone loss.

## 4. Materials and Methods 

### 4.1. Preparation of Fermented Oyster Extract (FO)

Fermented oyster extract was obtained from Marine Bioporcess Co. Ltd. (Busan, Republic of Korea) and extracted as described previously [33]. In brief, deshelled and frosted Pacific oysters (*Crassostrea gigas*) purchased from Deokyeon Seafood Co. Ltd (Tongyeong, Republic of Korea) were washed with tap water, homogenized (Han Sung Pulverizing Machinery Co. Ltd, Gyeonggi, Republic of Korea), and hydrolyzed with alcalase (Alcalase^®^ 2.4L FG, Brenntag Korea Co., Ltd., Seoul, Republic of Korea) at 60 ± 5 ℃ for 4 h. The hydrolyzed oyster was filtered using a vibrating sieve (120 mesh, BÜCHI Labortechnik GmbH, Essen, Germany) and diatomite filter press, and concentrated (brix 14) by rotary evaporator (BÜCHI Labortechnik GmbH, Essen, Germany). For FO manufacturing, Lactobacillus brevis BJ20 (Accession No. KCTC 11377BP) was inoculated into sterile seed media containing 3% yeast extract, 1% glucose, 1% monosodium glutamate, and 95% water. Seed media cultured at 37 ℃ for 24 h was inoculated at 10% (*v*/*v*) into the culture media (4% yeast extract, 1% glucose, 6% monosodium glutamate, 42% hyrolyzed oyster extract, and 47% water) and then sterilized, fermented at 37 ℃ for 48 h, filtered by filter press (Korea Filter, Daejeon, Republic of Korea), concentrated and spray dried to obtain an FO powder sample. 

### 4.2. Reagents and Antibodies

Recombinant M-CSF and RANKL were purchased from R&D Systems (Minneapolis, MN, USA). Antibodies against phospho-JNK, JNK, phospho-ERK, ERK, phospho-p65, and phospho-IκBα were obtained from Cell Signaling Technology (Danvers, MA, USA). The antibody against NFATc1 was purchased from BD Pharmingen (San Diego, CA, USA). Fetal bovine serum (FBS) and α-minimum essential medium (α-MEM) were obtained from Gibco BRL (Grand Island, NY, USA).

### 4.3. Animals and Treatments

To determine the effect of fermented oyster extract (FO) on osteoporosis in vivo, female ICR mice (8 weeks old) were purchased from Dae Han Bio Link (Chungbuk, Republic of Korea) and acclimated for 1 week, as described previously [34]. All animal experiments were approved by appropriate committees on the care and use of animals in research at Kyungpook National University and were conducted in accordance with established guidelines for the care and use of laboratory animals (KNU 2017-57). The acclimatized mice were randomly assigned to the following five groups: sham-operated with vehicle (Sham), surgically ovariectomized (OVX) with vehicle (OVX + V), OVX with 17β-estradiol (E2, 10 μg/kg) (OVX + E2), OVX with low concentration of FO (OVX + FO 100 mg/kg), and OVX with high concentration of FO (OVX + FO 200 mg/kg). FO was orally administered in distilled water for 4 weeks, and the same volume of distilled water was given as a vehicle control in the Sham and OVX +V groups. Mice in the positive control group (OVX + E2) were intraperitoneally injected with E2 at a dose of 10 μg/kg daily [35,36]. At the end of the treatment period, the mice were fasted and anesthetized, and the long bones (femurs and tibias) and blood were collected for bone morphometric and serum biochemical analysis, respectively. No significant adverse events were observed.

### 4.4. Micro-CT and Histomorphometric Analysis

Bone morphometric parameters of formaldehy-fixed tibias were measured using high-resolution micro-computed tomography (μCT, Skyscan 1272; Kontich, Belgium) with a source voltage of 60 kV, current of 166 μA and resolution of 8 μm. Bone volume per total volume (BV/TV), bone mineral density (BMD), trabecular separation (Tb. Sp.), and trabecular number (Tb. N.) were evaluated using CTAn software (Bruker; Kontich, Belgium) as reported previously [37,38]. Three-dimensional bone structure images were generated using CTAn software (Bruker; Kontich, Belgium).

For histomorphometric analysis, fixed tibias were decalcified in 12% EDTA and embedded in paraffin. Tissue sections were prepared using a microtome (Leica Biosystems, Nussloch, Germany) for hematoxylin and eosin (H&E) and tartrate resistant acid phosphatase (TRAP) staining. The number of osteoclasts per bone perimeter (N. OC/B. Pm) was assessed in sections stained for TRAP [39].

### 4.5. Osteocalcin and CTX-1 Measurements

Serum osteocalcin as a biomarker of bone formation was measured with a Mouse Osteocalcin EIA kit (Biomedical Technologies, Stoughton, MA, USA) according to the manufacturer’s instructions. Serum levels of C-terminal telopeptide fragment of type I collagen C-terminus (CTX), which is generated by osteoclasts as a biomarker of bone resorption, were evaluated using the RatLaps ELISA kit (Nordic Bioscience Diagnostics, Herlev, Denmark).

### 4.6. In Vitro Osteoclastogenesis Assay

Bone marrow-derived macrophages (BMMs) were prepared from bone marrow cells obtained from the tibias and femurs of 8-week-old mice (Dae Han Bio Link; Chungbuk, Republic of Korea) as described previously [38]. In brief, bone marrow cells were cultured in α-minimum essential medium (α-MEM) containing 10% fetal bovine serum (FBS). The next day, floating cells were collected and incubated in α-MEM supplemented with 10% FBS and M-CSF (30 ng/mL) to obtain bone marrow-derived macrophages (BMMs). After 3 days, BMMs were cultured with 20 ng/ml RANKL and 10 ng/ml M-CSF in the presence or absence of FO (0, 200, 400, or 600 μg/mL). The culture medium was replaced every other day. At the end of the culture period, the cells were fixed in fixative solution (a combination of 25 mL citrate solution, 65 mL acetone, and 8 mL of 37% formaldehyde) for 1 min and stained for TRAP activity using an Acid Phosphatase, Leukocyte (TRAP) staining kit (Sigma–Aldrich, St. Louis, MO, USA). TRAP-positive multinucleated cells (MNCs, ≥3 nuclei) were regarded as osteoclasts and counted.

### 4.7. Pit Formation Assay

BMMs were seeded on bone slices (IDS Nordic Bioscience, Herlev, Denmark) and cultured in osteoclast-inducing media containing 10 ng/mL M-CSF and 20 ng/mL RANKL. FO (600 μg/mL) or vehicle was added after 3 days of culture as described previously [40]. Cells attached to bone slices were removed by incubation with 1N NaOH for 20 min and washed with PBS. The bone slices were stained with Mayer’s hematoxylin to visualize resorption pits. The resorption area was quantified using the i-Solution image analysis program (IMT i-Solution; Daejeon, Republic of Korea).

### 4.8. Quantitative PCR Assay

Real-time PCR was performed as reported previously [41]. Briefly, total RNA was extracted using TRI-solution (Bioscience; Seoul, Republic of Korea) following the manufacturer’s instructions. Single-stranded cDNA was synthesized from 1 μg of total RNA using SuperScript II Reverse Transcriptase (Invitrogen, Carlsbad, CA, USA). We performed real-time PCR using a LightCycler 1.5 real-time PCR system (Roche Diagnostics, Basel, Switzerland) and the SYBR Premix Ex Taq (Takara Bio Inc., Shiga, Japan). The primer sequences used were as follows: TRAP (*Acp5*), 5′-TCCCCAATGCCCCATTC-3′ and 5′-CGGTTCTGGCGATCTCTTTG-3′; Cathepsin K (*Ctsk*), 5′-GGCTGTGGAGGCGGCTAT-3′ and 5′-AGAGTCAATGCCTCCGTTCTG-3′; *Dcstamp*, 5′-CTTCCGTGGGCCAGAAGTT-3′ and 5′-AGGCCAGTGCTGACTAGGATGA-3′; *Nfatc1*, 5′-ACCACCTTTCCGCAACCA-3′ and 5′-TTCCGTTTCCCGTTGCA-3′.

### 4.9. Western Blot Assay

Western blot was performed as reported previously [42]. Briefly, cells were treated with RIPA buffer containing phosphatase and protease inhibitors (Roche Applied Science, Mannheim, Germany). Protein concentration was determined using a bicinchoninic acid (BCA) assay (Pierce, Rockford, IL, USA), and equal amounts of protein were separated by 10% SDA-PAGE and transferred to nitrocellulose membranes (Whatman, Florham Park, NJ, USA). The membranes were blocked with 3% skim milk in TBS-T (TBS with Tween-20) at room temperature for 1 h and subsequently incubated with target primary antibodies (1:1000 dilution) at 4 °C overnight. The blot was then incubated with the appropriate horseradish peroxidase-conjugated secondary antibodies (1:2000 dilution) for 1 h. After washing the membranes with TBS-T, they were incubated with an enhanced chemiluminescence (ECL) reagents (Advansta, Menlo Park, CA, USA) for 1 min and images were acquired using a chemiluminescence imager (Azure Biosystems, Inc., Dublin, CA, USA).

### 4.10. Immunofluorescence Staining

BMMs cultured on glass coverslips with 10 ng/mL M-CSF and 20 ng/mL RANKL in the presence or absence of FO (600 μg/mL) were fixed with 4% paraformaldehyde for 15 min and then permeabilized with 0.2% Triton X-100. After blocking with 3% bovine serum albumin (BSA) in PBS, cells were incubated with anti-NFATc1 antibody followed by Alexa Fluor-488 conjugated anti-mouse secondary antibody. Cells were then stained with rhodamine-conjugated phalloidin (Cytoskeleton, Denver, CO, USA) and DAPI (Santa Cruz Biotechnology, Santa Cruz, CA, USA).

### 4.11. Statistical Analysis

Data are presented as the mean ± standard deviation (SD) of three independent experiments. Statistical analysis was performed by two-tailed Student’s t-test or one-way analysis of variance (ANOVA) with Tukey’s multiple comparison post-hoc test. * *p*-values < 0.05 were regarded as significant.

## Supplementary Materials

The following are available online at https://www.mdpi.com/2072-6643/11/6/1392/s1, Figure S1: Effect of FO on uterine index in OVX mice, Figure S2: Effect of FO on osteoclastic resorption activity.
