# Supplementary material for: Fermented Oyster Extract Prevents Ovariectomy-Induced Bone Loss and Suppresses Osteoclastogenesis
Source: Nutrients. 2019 Jun 21;11(6):1392. doi: 10.3390/nu11061392 (PMC6627411; doi:10.3390/nu11061392)
Supplement: Supplementary file 1 [file nutrients-11-01392-s001.pdf]

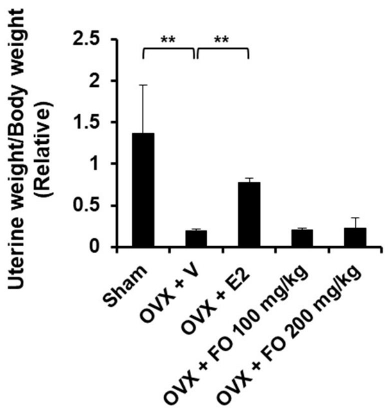

Figure S1. Effect of FO on uterine index in OVX mice. The relative uterine index was assessed as uterine weight divided by body weight. Sham, Sham-operated with vehicle; OVX + V, OVX with vehicle; OVX + E2, OVX with 17β-estradiol (E2, 10 μg/kg); OVX + FO 100 mg/kg, OVX with low concentration of FO; OVX + FO 200 mg/kg, OVX with high concentration of FO. Values are the mean ± SD (n = 8). ** $p < 0.01$.

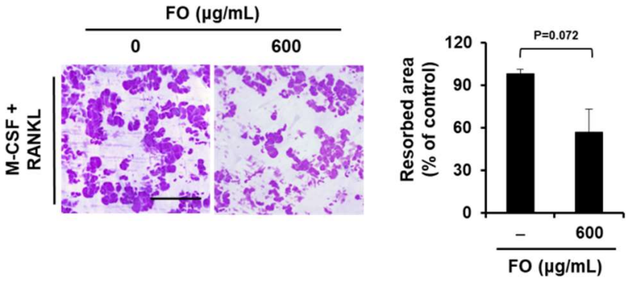

Figure S2. Effect of FO on osteoclastic resorption activity. BMMs plated on bone slices were cultured in osteoclast-inducing medium. After 3 days, the cells were treated with FO (600 μg/ml) or vehicle for 24 h. The resorption areas were quantified using the i-Solution program (right graph).
